# Exercise and Health: Can Biotechnology Confer Similar Benefits?

**DOI:** 10.1371/journal.pmed.0020068

**Published:** 2005-03-29

**Authors:** R. Sanders Williams, William E Kraus

## Abstract

Education and public policies are largely failing to encourage people to exercise. Could our knowledge of exercise biology lead to pharmaceutical treaments that could confer the same benefits as exercise?

## Health Benefits of Physical Activity

Regular physical activity has been recognized to confer health benefits since antiquity [1]. However, for most of humankind, voluntary discretion over whether or not to exercise is a recent phenomenon limited to advanced industrialized societies.

A large body of epidemiological literature consistently documents greater longevity in persons who are physically active on a near-daily basis, and reveals inverse relationships between levels of daily exercise and incidence of major chronic disorders such as obesity [2], hypertension [3], diabetes [4], ischemic heart disease, and all causes of mortality [5,6,7,8,9,10,11,12]. From a public health perspective, there is little question that even modest increases in daily activities such as walking or stair climbing would have important positive consequences in reducing the burden of illness.

However, knowledge of the likely health benefits accruing to the physically active so far has not been a sufficient stimulus to promote sustained changes in behavior for most of the American population. If education and public policies are insufficient to promote behavioral changes to increase physical activity among most people, can advances in biotechnology confer such benefits to individuals unable or unwilling to perform the necessary physical effort?

## Translating Knowledge of Exercise Biology to Novel Therapeutics

Greater knowledge of how cells and tissues are modified in response to recurring bouts of exercise provides a basis for more precise recommendations as to the mode, intensity, and amount of exercise required to produce specific health benefits (e.g., treatment of dyslipidemia [13], control of body weight [14], or prevention of diabetes [15]). In addition, an understanding of the molecular signaling events that drive the beneficial effects of exercise on human physiology could foster the development of novel drugs, devices, or biological agents designed to substitute for exercise.

Many individuals who otherwise would develop diabetes or cardiovascular disease would benefit if advances in exercise biology revealed novel measures to promote the favorable effects on insulin sensitivity, lipoprotein metabolism, and blood pressure that are known to accrue through regular physical activity.

## Physiological Properties of Skeletal Muscle

**Figure 1:**
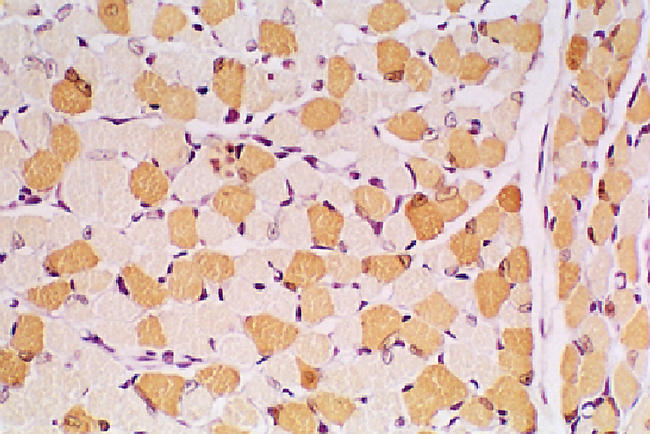
Specialized Myofibers in a Mammalian Skeletal Muscle A cross-section of the gastrocnemius muscle of a mouse has been stained to detect myoglobin, which is found selectively in slow oxidative and fast oxidative myofibers (stained brown), but not in fast glycolytic myofibers (unstained). Human muscles exhibit a similar mosaic pattern. In response to sustained periods of motor nerve stimulation repeated daily for several weeks, the percentage of myofibers that contain myoglobin is increased, in synchrony with an increased abundance of mitochondria and a shift of myosin subtypes from fast glycolytic to slow or fast oxidative.

What do we know about basic muscle and exercise biology? The cells that constitute our skeletal muscles are called myofibers—large multinucleated cells that may extend for the full length of individual muscles. There are different types of myofibers, which vary in size and with respect to metabolic and contractile capability [16] (Figure 1). Skeletal myofibers are innervated by motor neurons that contact each myofiber, and the intensity, duration, and timing of each muscle contraction are determined by the pattern of motor neuron firing. A pattern of occasional intense contractions separated by longer periods of rest is called “phasic,” while a pattern characterized by brief contractions occurring repeatedly over an extended period is called “tonic.” Endurance training regimens like running or cycling employ tonic patterns of contractile work, and it is this form of habitual activity that serves best to reduce risk for obesity, diabetes, hypertension, and heart disease.

## Dynamics of Muscle Mass

Maintenance of normal muscle mass requires some minimal level of ongoing work activity, and building and maintaining muscle mass is most effectively done through phasic contractions. A slow but inexorable loss of muscle mass is a feature of advancing age in human populations [17]. Loss of muscle mass and strength is an important determinant of injury and disability in the elderly, but even rigorous weight training programs cannot completely counteract this age-related decline that becomes particularly troublesome in the eighth and ninth decades of life. Efforts to develop effective countermeasures to maintain muscle mass in the elderly constitute an active and important area of current research [18,19,20].

Although the molecular signaling mechanisms that transduce the effects of phasic patterns of work activity to modify muscle mass are incompletely understood, recent evidence implicates pathways that include the signaling molecules PI3 kinase, Akt, mTOR, S6K, and ERK, the ubiquitin ligases MAFbx and MuRF1, and transcription factors of the FOX superfamily in the control of both catabolic and anabolic processes [21,22,23,24].

## Contractile and Metabolic Properties

With respect to variations in contractile and metabolic properties, myofibers are classified on a spectrum between two extremes on the basis of contractile (fast versus slow) and metabolic (glycolytic versus oxidative) properties. At one extreme, the fastest glycolytic fibers have high levels of enzymes that generate ATP via glycolysis but few mitochondria (approximately 1% of cell volume). At the other end of the spectrum, slow oxidative fibers generate force with slower kinetics but are capable of long periods of repeated contraction without fatigue. They are rich in mitochondria (3%–10% of cell volume). Other myofibers, called fast oxidative, are both relatively fast and resistant to fatigue, and are rich in mitochondria (like the slow oxidative fibers). Muscles composed primarily of fast glycolytic fibers are needed for rapid movements (e.g., escape from predators) but fatigue when sustained periods of activity are required (e.g., migration).

Most human muscles exhibit a mosaic pattern of different fiber types (Figure 1), with a great deal of variation among individuals, which is influenced at least in part by patterns of use. When we exercise daily, or at least several times weekly, we deliver a stimulus to the specific muscle groups involved in these activities that is sufficient to alter specialized properties of myofibers within these muscles. While habitual physical activity promotes a great variety of physiological adaptations that alter vascular reactivity, cardiac function, adipocyte function, and neurophysiology, adaptive responses of skeletal myofibers confer at least some of the health benefits.

Patterning of skeletal muscle fiber composition is initially determined during embryonic development, but can be partially or completely overturned by stimuli applied to fully mature adult myofibers: by hormonal influences (e.g., thyroid hormone), but most importantly by different patterns of motor nerve activity and contractile work. Myofibers that experience phasic patterns of contractile work—brief bursts of activity interspersed within long periods of inactivity—will assume the fast glycolytic phenotype. Myofibers subjected to tonic patterns of work activity—sustained periods of repetitive contraction on a habitual basis—will take on fast oxidative or slow oxidative properties. Under experimental conditions in laboratory animals, it is possible to transform muscles completely from one myofiber phenotype to another in a reversible manner, solely by altering the pattern of neural stimulation. We know that having a high proportion of oxidative muscle fibers conveys health benefits, and the possibility to control fiber composition through therapeutic intervention is promising.

## Molecular Signaling Pathways

**Figure 2:**
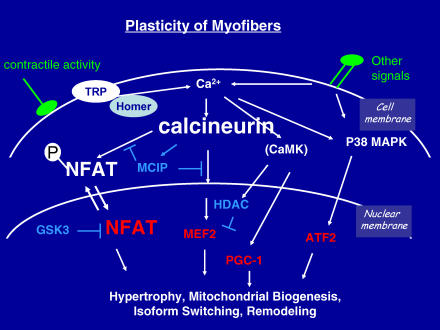
Molecular Signaling Pathways Link Changes in Contractile Activity to Changes in Gene Expression That Establish Myofiber Diversity A tonic pattern of motor nerve activity promotes changes in intracellular calcium that trigger a variety of intracellular events that modify the function of nuclear transcription factors. The pathway transduced by calcineurin and NFAT is highlighted in larger type. Other signals are received by cell surface receptors to activate similar or parallel signaling events. Signaling proteins that participate in transducing effects of contractile activity to specific genes include ion channels (TRP), scaffolding proteins (Homer), protein phosphatases and protein kinases (calcineurin, CAMK, p38MAPK), DNA-binding transcription factors (shown in red; NFAT, MEF2, PGC-1, ATF2), and endogenous inhibitors (shown in blue; GSK3, HDAC, and MCIP) (inhibitors antagonize gene activation via the pathways indicated, in some cases acting as negative feedback regulators).

At a cellular and molecular level, how does a fast glycolytic myofiber sense a tonic pattern of contractile activity and transduce that information to transform itself into a cell with fast oxidative or slow oxidative properties? We know that such signals must be transduced to the nucleus, activating certain genes and suppressing others, for myofiber plasticity to occur. We know the identities of some of the nuclear transcription factors that carry these signals, and of other proteins that regulate the function of these transcription factors (Figure 2).

Quite a variety of intracellular messengers have been proposed to provide the proximate signals in exercising muscles to stimulate activity-dependent gene regulation. This discussion will focus on a signaling cascade mediated by calcineurin, a calcium-regulated protein phosphatase that signals to the nucleus via transcription factors of the nuclear factor of activated T cells (NFAT) family. Upon receipt of the appropriate calcium signal, calcineurin is activated and removes phosphate groups from NFAT, thereby permitting translocation of NFAT to the nucleus. Within the nucleus, NFAT binds DNA and activates transcription (in concert with other transcription factors) of relevant downstream target genes that encode proteins necessary for fast oxidative or slow oxidative myofiber phenotypes.

Calcineurin and NFAT proteins are abundant in skeletal myofibers, and several lines of evidence support the viewpoint that the calcineurin–NFAT pathway plays a role in mediating activity-dependent gene regulation in muscle [25,26,27,28,29,30,31,32,33,34,35,36,37,38,39]. For example, in mice genetically engineered to distinguish the inactive (cytoplasmic) from active (nuclear) forms of NFAT by means of a sensor, it is evident that NFAT is inactive in resting muscles, but activated by tonic patterns of muscle contraction (running or electrical stimulation of the motor nerve) [40]. Using other genetic manipulations in mice to produce in muscle a form of calcineurin that remains active even in the absence of calcium signals, myofibers are converted from fast glycolytic to fast oxidative or slow oxidative forms [41]. And in muscles of mice genetically engineered to lack calcineurin, fiber type switching is impaired [42].

## Cellular Memory

**Figure 3:**
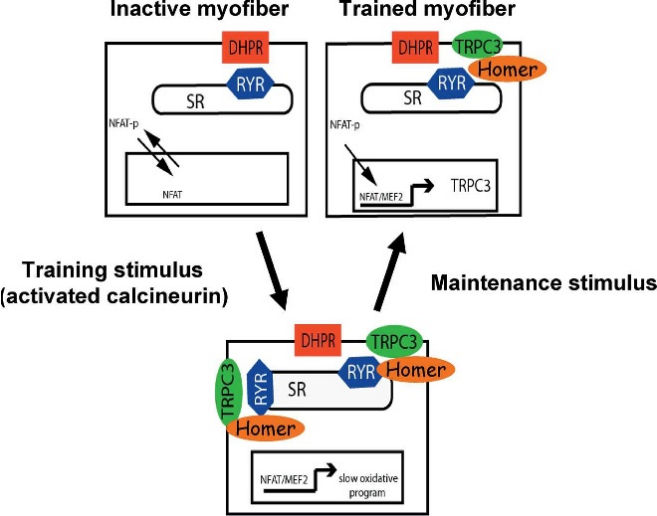
Proposed Model for Cellular Memory, Based on Activity-Induced Changes in TRPC3—A Putative Store-Operated Calcium Channel Neural activation triggers muscle contraction by releasing calcium stored within the sarcoplasmic reticulum (SR) through mechanisms that involve channel proteins called dihydropyridine receptors (DHPR) and ryanodine receptors (RYR). Inactive myofibers have a low abundance of TRPC3 channels, and calcium released from SR is not sufficient to maintain the calcium-regulated transcription factor NFAT in the nucleus. Under conditions of tonic activity (training stimulus), TRPC3 channels become more abundant, and are regulated by the scaffold protein Homer, which binds RYR. Under these conditions, the combined effect of calcium entering the cell via TRPC3 channels and exiting the SR via RYR channels maintains NFAT in the nucleus, where it promotes transcription of genes that establish the slow oxidative phenotype in myofibers. Once the slow oxidative phenotype is established (trained myofiber), the continued expression of TRPC3 allows this state to be maintained even with a less intensive tonic activity pattern of neural stimulation. (Figure adapted from [40].)

Muscle contractions are initiated under the influence of the motor nerve by release of calcium from the sarcoplasmic reticulum, which triggers actin–myosin crossbridge cycling (Figure 3). Calcium released via ryanodine receptors is completely sufficient to activate muscle contractions, and the effects are immediate (within milliseconds). It is also sufficient to initiate calcineurin–NFAT signaling to the nucleus, but cannot by itself sustain the signal in a manner necessary to promote myofiber remodeling [40]. Changes in gene expression evoked by neuromuscular activity are not immediate but require that the stimulus be sustained for an extended period (minutes to hours). Moreover, tonic stimulation of the motor nerve must be repeated daily, or nearly so, over several weeks for the changes in myofiber properties to become fully manifest. We have characterized this requirement for repetition of the activity stimulus over days as a form of “cellular memory.” The effects of the tenth or 20th day of exercise are not the same as the effects of the first day. The myofiber somehow “remembers” not only the pattern of activity it has experienced today, but what has gone on over the preceding days or weeks, such that the changes in abundance of proteins that control contractile function and metabolism accrue over time.

To explain this cellular memory, we propose that, as the bursts of contractile activity are sustained over time (through a tonic pattern of neural stimulation), a second source of calcium is mobilized from outside of the cell and enters via a class of calcium channels that are called “store-operated” or “non-voltage-dependent.” This second source of calcium is not required for muscle contractions, but is required to sustain calcium-dependent signaling to the nucleus. Phasic patterns of contractile activity do not promote calcium entry via store-operated channels. Tonic patterns of activity, in contrast, would not only promote the mobilization of extracellular calcium but also increase the number of store-operated calcium channels with each bout of exercise. Myofibers would thereby grow progressively more responsive to tonic activity. Consistent with this model, we know that daily running increases the expression of a putative store-operated calcium channel called TRPC3. Moreover, increasing the abundance of TRPC3 in cultured myotubes prolongs the period in which intracellular calcium is elevated following a depolarizing stimulus, sustains the transcription factor NFAT within the nucleus, and augments expression of NFAT-dependent target genes [40].

A great deal of additional research remains to be done before we have a comprehensive understanding of how habitual physical activity promotes changes in gene expression in skeletal muscles, and in turn improves fitness and reduces risk for diabetes, hypertension, dyslipidemia, and coronary artery disease. However, studies of the relationships between the proteins of calcium metabolism and calcium-regulated signaling pathways—as described here in a simplified manner with respect to TRPC3, calcineurin, and NFAT proteins—are illustrative of progress in this field. Other notable findings point to additional signaling proteins (CAMK, p38MAPK, and AMPK) and transcription factors (PGC-1, MEF2, ATF2, PPARs) active in pathways that intersect with calcineurin–NFAT signaling [31,43,44,45,46,47,48] (see Figure 2). It is encouraging that some of these proteins are attractive targets for drug discovery.

## Summary and Conclusions

Long the province of physiologists who have contributed valuable insights in past decades, exercise science more recently has attracted the attention of molecular biologists, who have recognized the biological interest and medical importance of this field. Biotechnology and pharmaceutical companies also are beginning to take interest.

This review has focused on adaptive responses of skeletal muscle to changing patterns of physical activity, and on the role of the calcium–calcineurin–NFAT signaling cascade in controlling gene expression in skeletal myofibers. Further advances in our understanding of signaling mechanisms that govern activity-dependent gene regulation in skeletal muscle could lead to drugs, gene therapy, or devices that can, at least in part, substitute for daily exercise. Although it is unlikely that such technologies would fully recapitulate exercise-induced adaptations that affect other tissues of the body, beneficial effects on work performance and whole-body metabolism have been demonstrated using gene transfer techniques to alter skeletal muscles in animal models. If it proves possible to drive similar effects in skeletal muscles in humans, the interventions capable of providing such effects would almost certainly find broad clinical application.

## Abbreviation

NFAT: nuclear factor of activated T cells
